# Functional outcomes of single-session holmium laser enucleation of the prostate and high-intensity focused ultrasound in management of patients with prostate cancer and enlarged prostate: results from a pilot study

**DOI:** 10.1007/s00345-024-05424-0

**Published:** 2024-12-30

**Authors:** Jessica Delgado, Joao G. Porto, Ansh Bhatia, Adele Raymo, Ruben Blachman-Braun, Tarek Ajami, Aravindh Rathinam, Pedro F. S. Freitas, Archan Khandekar, Robert Marcovich, Dipen J. Parekh, Bruno Nahar, Hemendra N. Shah

**Affiliations:** 1 Desai Sethi Urology Institute, University of Miami Miller School of Medicine, 1120 NW 14th Street, Miami, FL 33136 USA; 2 Department of Interventional Radiology, University of Miami Miller School of Medicine, Miami, USA; 3 University of Miami Miller School of Medicine, Miami, USA; 4 Sylvester Comprehensive Cancer Center, Miami, USA

**Keywords:** BPH, HoLEP, HIFU, Prostate cancer

## Abstract

**Purpose:**

In patients with prostate cancer (PCa), focal therapy with High-Intensity Focused Ultrasound (HIFU) combined with benign prostatic hyperplasia (BPH) surgery has been used to improve immediate post-operative voiding symptoms. Our study aimed to evaluate the functional outcomes of patients undergoing simultaneous holmium laser enucleation of the prostate (HoLEP) + HIFU and compare them to those who underwent HoLEP for bladder outlet obstruction secondary to BPH.

**Methods:**

We performed retrospective review of patients who underwent HoLEP + HIFU or HoLEP between June 2017 and May 2024. The nearest neighbor method with age and prostate volume were used to propensity match HoLEP + HIFU patients with HoLEP only patients in a 1:2 ratio. Demographics, functional characteristics, and complications of patients who underwent HoLEP + HIFU were compared with patients undergoing only HoLEP for BPH.

**Results:**

A total of 99 patients were analyzed, of which 33 patients underwent combined HIFU with HoLEP. Patients undergoing HIFU + HoLEP experienced higher rates of acute urinary retention (*p* = 0.016) and transient urinary incontinence, along with a delayed recovery of full continence, compared to those who underwent HoLEP alone. Postoperative urinary tract infection (UTI), urethral stricture, bladder neck stenosis (BNS), and continence rate were similar between the groups.

**Conclusion:**

Patients undergoing HoLEP + HIFU seems to have a higher risk of post-operative acute urine retention and delayed recovery from transient urinary incontinence, compared to HoLEP alone. The addition of HIFU to HoLEP does not influence the rate of UTI, urethral stricture, BNS, or improvement of voiding parameters up to one year follow up.

## Introduction

The need to reduce the morbidity associated with traditional whole-gland treatment for prostate cancer (PCa), combined with the advancement of imaging technologies improving disease detection, has driven a shift in the choice of treatments for localized PCa toward focal therapies (FT). Among these, high-intensity focused ultrasound (HIFU) has gained interest as an effective minimally invasive treatment for localized PCa, being approved for prostate ablation in 2015 by the United States Food and Drug Administration (FDA) [1]. It has also garnered interest as an effective treatment with acceptable oncologic control [2].

However, challenges associated with large prostates and/or prostatic have been identified, as they may compromise the precision of energy transmission to the targeted tissue [3]. Additionally, postoperative complications such as prostatic edema-induced acute urinary retention (AUR) requiring prolonged indwelling urethral catheterization, as well as the development of urethral strictures and fistulas, have been reported following HIFU monotherapy [1, 4–6]. To improve functional outcomes and mitigate HIFU-related obstruction and lower urinary tract symptoms (LUTS), combination therapy with surgical benign prostatic hyperplasia (BPH) modalities has been recommended [4, 5].

Current evidence supports the use of combined transurethral resection of the prostate (TURP) and HIFU as standard technique for focal ablative therapy in localized PCa [7]. Improvement of HIFU-related treatment outcomes, reduction in duration of urethral catheterization, and improved urinary status are all well-documented advantages of adjunct TURP [7]. However, the evolution of endoscopic BPH management has introduced modalities that may rival TURP in this setting. Among these, holmium laser enucleation of the prostate (HoLEP) has demonstrated several advantages, including a lower blood transfusion rate, shorter catheterization duration time, and hospital stay when compared to traditional TURP. According to American Urology Association guidelines, HoLEP should be considered as a prostate size-independent options for the treatment of BPH and specifically for those at higher risk for bleeding such as patients on anticoagulant therapy [8, 9]. Considering efficacy and superior outcomes, HoLEP can potentially replace TURP as the standard of care in combined therapy by overcoming limitations associated with focal HIFU, reducing postoperative morbidities, and improving LUTS.

Despite promising individual outcomes, data on the combination of HoLEP and focal HIFU remain sparse, with existing studies largely focusing on non-synchronous procedures and excluding patients with prostates > 50 g. Preliminary findings suggest improved functional outcomes with HIFU + HoLEP compared to HIFU monotherapy [4]. Building on our prior work demonstrating the safety and feasibility of combining HoLEP and HIFU for patients with prostates > 60 g [10], the present study aims to evaluate the safety, efficacy, and functional outcomes of simultaneous HoLEP + HIFU for patients eligible for focal HIFU with concomitant BPH. By comparing these outcomes to those of patients undergoing HoLEP for bladder outlet obstruction alone, we seek to provide evidence that can enhance preoperative counseling and inform patient selection for this novel combined approach.

## Methods

Following the approval of the Institutional Review Board (20180511), we conducted a retrospective analysis of 710 patients who underwent HoLEP at our institution between July 2017 and May 2024 to identify patients with localized PCa who also received HIFU FT in the same session of HoLEP (study arm). These patients were matched-paired (1:2) with patients undergoing HoLEP for BPH (control arm). Matching was based on age, prostate size, and anticoagulant usage and was conducted using the nearest-neighbor method. Patients with associated neurogenic disorders, urethral stricture, bladder cancer, or a history of locally advanced PCa were excluded from both groups in the present study.

All patients with localized PCa who opted for HIFU after shared making were considered for combined HoLEP if their prostate volume was > 60 g or they had any kind of obstructive symptoms secondary to bladder outlet obstruction. The HoLEP procedures were executed using the “En-bloc” technique, utilizing a 550 μm Ho: YAG fiber and a 26 Fr laser endoscope (Karl Storz, Tuttlingen, Germany) with 60 W energy settings. Morcellation was conducted using the VersaCut Tissue Morcellator or Piranha Morcellator. Following HoLEP, those with localized PCa underwent Focal HIFU using either the Ablatherm^®^ HIFU machine (EDAP TMS, France) or the Focal One^®^ device (EDAP TMS, France). All patients had hemi-gland ablation and were admitted for overnight monitoring post-procedure. They underwent continuous bladder irrigation with 0.9% normal saline. Bladder drainage was managed with a 20–22 Fr Foley catheter off traction and removed on the first postoperative day before discharge. BPH medication was stopped postoperatively. All patients were followed up at six weeks and 3, 6, and 12 months postoperatively. All patients in both groups had prostate-specific antigen (PSA) at 3 months to look for nadir level post-HoLEP. Data collected included demographic, perioperative, and functional characteristics, with complications assessed using the Clavien-Dindo classification [11]. Postoperative continence rates were collected, and incontinence was defined as the presence of involuntary urine leakage of any severity needing protection or pads at any time postoperatively. Patients with postoperative incontinence were managed with a combination of lifestyle modifications and Kegel exercises. Few patients were given anticholinergic medications at their 6-week postoperative follow-up visit, based on clinical diagnosis of overactive bladder with predominant urge incontinence. We collected the International Prostate Symptom Score (IPSS), maximum urinary flow rate (Qmax), and post-void residual volume (PVR) at baseline and at 3-, 6-, and 12 months post-surgery. Medical and surgical retreatment for BPH related to LUTS were noted.

The patient’s demographic data, baseline characteristics, perioperative outcome, and postoperative outcome were assessed and compared between both groups to ensure comparable populations. Shapiro-Wilks test and the visual plots were used to assess normality. Continuous variables were expressed as mean ± standard deviation if parametric and median (IQR) if non-parametric or questionnaires. Categorical variables were summarized as counts and percentages. The unpaired t-test was used to compare parametric variables between groups, and the Wilcoxon signed Rank test was employed to compare outcomes between non-parametric variables, as appropriate. Comparisons between subgroups were assessed using the Chi-square test (and Fischer’s test, when applicable) for categorical variables. A two-sided *p*-value < 0.05 was considered statistically significant. All statistical analysis was performed in RStudio version 2023.09.01 (RStudio Inc, MA, USA).

## Results

A total of 710 patients underwent HoLEP at our institution between July 2017 and May 2024. Of these 33 patients with localized PCa received HIFU FT in the same session of HoLEP (study arm). These patients were matched-paired (1:2) with 66 patients undergoing HoLEP for BPH (control arm). Patients in the study group, consisting of those with PCa, exhibited significantly higher preoperative PSA levels (*p* < 0.001) and PSA density (*p* = 0.002) compared to the control group (Table 1). The control group, however, had worse baseline urinary parameters, including a significantly higher median IPSS (17 vs. 10, *p* = 0.005), lower median Qmax (6.4 vs. 9.5 ml/sec, *p* = 0.028), and higher PVR (210 vs. 62 ml, *p* = 0.002) (Table 2). The post-HoLEP prostate volume was available for 24 patients. The mean baseline prostate volume of these patients was 113.3 ± 58.24 g and the post-HoLEP prostate volume as measured by FocalOne was 33.2 ± 11.8 g. There was 82.2 ± 59.9 g (72.4%) reduction in prostate volume after HoLEP. The prostate volume as measured by FocalOne also include the volume of prostatic fossa (cavity) that is created by enucleation.

**Table 1:** Comparison of clinical, demographic, and biochemical characteristics between groups

	HoLEP (66)	HoLEP + HIFU (33)	*p*-value
**Age** (years)	72.92 ± 7.25	73.4 ± 6.69	0.734
**BMI** (Kg/m^2^)	27.63 ± 3.74	28.23 ± 3.81	0.462
**Race/ethnicity**			
White or Caucasian (%)	56 (84.8%)	27 (81.81%)	0.543
Black/African American (%)	4 (6.1%)	4 (12.12%)	
Asian (%)	0	0	
Multiracial/not reported (%)	6 (9.1%)	2 (6.07%)	
**Preoperative PSA** ng/mL	4 [2.2–6.7]	6.9 [5.73–10.8]	**< 0.001**
**Baseline PV** (g)	95.5 [66.75–138.7]	100.46 [75–130]	0.55
**Preoperative PSAd**	0.040 [0.023–0.072]	0.072 [0.051–0.11]	**0.002**
**Resected PV** (g)	80.6 [48.2–109.5]	81 [42–102]	0.86

BMI: Body Mass Index; HIFU: High-intensity focused ultrasound; HoLEP: Holmium Laser Enucleation of the Prostate; PV: Prostate Volume; PSA: Prostate-specific Antigen; PSAd: Prostate-specific Antigen density. Mean ± standard deviation, median [IQR 25th -75th ]

**Table 2:** Comparison of IPSS, Qmax, and PVR at baseline, 3 months, 6 months, and 1 year

Time	*N* (HoLEP; HoLEP + HiFU)	HoLEP	HoLEP + HIFU	*p*-value
IPSS				
Baseline	52; 22	17 [11–23]	10 [8–17]	**0.005**
3 months	45; 14	1 [0–4]	2 [2–5]	0.14
6 months	28; 12	0.5 [0–5]	2.5 [1.75–3]	0.253
12 months	20; 9	1 [0–4]	2 [0–3]	0.353
Qmax				
Baseline	35; 6	6.4 [4.3–9.15]	9.5 [9.32–11.1]	**0.028**
3 months	41; 14	15 [11–24]	16.5 [11.6–18.62]	1
6 months	29; 10	15 [12–24]	22.55 [19.15–22.02]	0.079
12 months	26; 12	18.1 [11–28.07]	20.85 [16.07–29.85]	0.649
PVR				
Baseline	55; 17	210 [85.5–356]	62 [29–100]	**0.002**
3 months	47; 15	30 [2.5–57]	15 [0–48]	0.451
6 months	33; 12	21 [5–66]	0 [0–6.5]	**0.003**
12 months	26; 40	11.5 [0–32]	23 [0–27]	0.890

HIFU: High-intensity focused ultrasound; HoLEP: Holmium Laser Enucleation of the Prostate; IPSS: International Prostate Symptom Score; PVR: Post-void residual volume; Qmax: Maximum urinary flow rate. Median [IQR 25th -75th ]

Postoperatively, the duration of catheterization and hospital stay did not significantly differ between the groups. However, adding HIFU led to a significantly higher rate of AUR (*p* = 0.016). Rates of postoperative complications such as UTIs (*p* = 1), urethral strictures (*p* = 1), and bladder neck stenosis (BNS) (*p* = 0.33) were comparable between the groups. Similarly, continence rates at the 6-week follow-up were similar (*p* = 0.137), although the combined therapy group took longer to regain continence, a difference that was not statistically significant (Table 3).

**Table 3:** Comparison of duration of catheter postoperative, length of stay, complication rate, and nadir PSA at 3 months between groups

	HoLEP (66)	HoLEP + HIFU (33)	*p*-value
**Catheter Duration** (days)	1 [1–1]	1 [1–1]	0.62
**Length of hospital stay** (days)	1 [1–1]	1 [1–1]	0.75
**Complications as per Modified Clavien- Dindo Classification**			
**Grade 1**			
AUR after TOV in the hospital (%)	2 (3.3%)	6 (10.8%)	**0.016**
Urinary Tract Infection (%)	3 (4.5%)	1 (3.03%)	1
TUI at 6 weeks (%)**	5 (7.9%)	3 (14.3%)	0.41
**Grade 2**			
Blood transfusions (%)	1 (2.3%)	0	1
TUI needing anticholinergics**	6 (9.5%)	4 (19%)	0.26
**Grade 3**			
Urethral Stricture (%)	1 (1.51%)	1 (3.03%)	1
Bladder Neck Stenosis (%)	0	1 (3.03%)	0.33
**Other relevant post-operative information**			
**PSA at 3-month**	0.37 [0.2–0.5]	0.39 [0.25–0.69]	0.72
Time to achieve continence (months)***	3.46 [1.57–6.86]	4.21 [2.73–10.1]	0.12

AUR: Acute Urinary Retention; HIFU: High-intensity focused ultrasound; HoLEP: Holmium Laser Enucleation of the Prostate; PSA: Prostate-specific Antigen; TOV: Trial of Void; TUI: transient urinary incontinence. Mean ± standard deviation, median [IQR 25th -75th ]. **- 63 + 21 patients analyzed;

Regarding short- and mid-term outcomes, both groups showed equal improvements in IPSS scores at 6 months (*p* = 0.253) and 12 months (*p* = 0.353) despite the baseline IPSS being higher in the HoLEP-monotherapy group (Table 2). Objective voiding parameters, such as Qmax and PVR, also improved significantly in both groups. Additionally, the median PSA nadir at 3 months postoperatively was similar between the control group (0.37 ng/dl) and the study group (0.39 ng/dl), with no significant difference (*p* = 0.72) (Table 3).

Moreover, no patients in either group required alpha-blockers or 5-alpha reductase inhibitors after surgery, nor did any patients need retreatment for BPH-related obstructive uropathy. Anticholinergic medications were required postoperatively in 6 patients in the study group and 4 in the control group. Although one patient from each group developed urethral strictures, and one patient from the study group developed BNS, all cases were successfully managed with holmium laser incision, and no recurrences were observed during follow-up (Table 3).

## Discussion

Historically, HIFU was recognized for its effectiveness in tissue destruction, making it a promising option for cancer treatment. However, it proved less effective in treating associated bladder outlet obstruction, as the resulting tissue shrinkage and necrosis often exacerbated the obstruction [12]. Both bladder neck obstruction and urethral strictures were the most common complications of HIFU treatment [13, 14]. Hence, patients with prostate > 30 g were given androgen deprivation therapy (ADT) to reduce prostate prior to HIFU. However, this strategy led to undesirable side effects from the hormonal treatment. TURP later emerged as an alternative to ADT, significantly reducing catheterization time and UTI incidence [13]. Consequently, combined TURP + HIFU became a frequently utilized multimodal treatment strategy to reduce postoperative voiding dysfunction [15]. However, the data shows that TURP prior to HIFU often failed to reduce intravesical obstruction completely and resulted in complications such as stenosis. Hatiboglu G et al. published outcomes associated with combined TURP or greenlight vaporization of the prostate performed 1 day prior to HIFU procedures in 131 patients with a mean prostate of 43.7 *±* 25.6 g [16]. They revealed no change in postoperative IPSS, Qmax, and PVR, and 21% of patients developed new-onset incontinence following the combined procedure. Unfortunately, 25% of patients needed additional surgical intervention for BNS. Similarly, Crouzet et al. noted a 16% incidence of bladder neck obstruction when TURP and HIFU were combined in one session [14]. However, the current data indicates that HoLEP may prove superior to TURP by mitigating postoperative complications, producing equivalent BPH and oncologic outcomes in patients with large adenomas (> 60 g), improved long-term functional outcomes, and fewer redo procedures than TURP [7, 10, 17].

Overall, we noted that adding HIFU did not negatively impact the duration of postoperative catheterization or hospital stay. It also did not affect functional voiding outcomes after HoLEP. There was no significant difference in the rates of postoperative UTI, urethral strictures, or BNS in patients who underwent HIFU + HoLEP when compared to HoLEP monotherapy. One negative impact of adding HIFU to HoLEP was the increased incidence of postoperative AUR and delayed recovery from transient urinary incontinence when compared with patients undergoing HoLEP alone. However, the continence rates were not statistically different amongst both groups. This evidence suggests that combining HoLEP + HIFU may be feasible for select PCa patients who want to treat their BPH surgically and associated LUTS. Unfortunately, there is no data in literature on outcome of combined HoLEP + HIFU to discuss with our results.

Additionally the variability in functional outcomes reporting after focal therapy remains a challenge. As noted in recent systematic reviews, functional outcomes of focal therapies like HIFU are often difficult to compare due to significant heterogeneity in reporting methodologies and follow-up strategies [18]. Earlier, we demonstrated good oncologic outcomes of combined HoLEP with HIFU for treating PCa [10]. We agree with the current literature stating that when combined with HIFU, HoLEP improves focal energy delivery by reducing prostate diameter and removing prostatic calcifications in the transitional zone, which may impede optimal tissue cavitation [19]. Our present study confirmed that adding HoLEP to HIFU improves voiding parameters without significantly increasing morbidity in patients with PCa. None of the patients in either group required medical or surgical retreatment for LUTS related to obstructing the prostate during the follow-up period. Both groups received anticholinergic medication at their first postoperative visit at 6 weeks. However, we did not collect data on postoperative anticholinergic drug use for detailed analysis in this study.

This study has a few limitations worth describing. First, this was a retrospective analysis of a prospectively maintained database from a single institution and a single HoLEP surgeon with short follow-up data at one year, limiting the generalizability of our outcomes. Our study did not compare the same setting outcomes with staged HoLEP + HIFU procedures. Additionally, we could not obtain continence outcomes for all patients, as many were international or from out of state and planned to continue follow-up care with their referring urologists. We tried contacting the referring physicians and patients directly to gather as much information as possible during the postoperative period. Nevertheless, our pilot study evaluated the outcomes of combined HoLEP + HIFU treatment for larger prostate volume than previously described. Our data confirms the safety and efficacy of combining HoLEP with focal HIFU in selected patients with localized PCa and large prostate volume.

## Conclusion

Combining HoLEP and HIFU in the same session offers a promising minimally invasive option for men with localized PCa and BPH. This approach significantly improved voiding parameters without increasing the risk of postoperative complications, such as UTI, urethral strictures, BNS, or long-term incontinence. However, patients receiving the combined treatment may experience a higher incidence of postoperative AUR and a delayed recovery of transient urinary incontinence.

## Data Availability

The raw data will be provided after approval of institution IRB.
